# The Neglected Pieces of Designing Collective Decision-Making Processes

**DOI:** 10.3389/frobt.2019.00016

**Published:** 2019-03-26

**Authors:** Yara Khaluf, Pieter Simoens, Heiko Hamann

**Affiliations:** ^1^IDLab, Ghent University-Imec, Ghent, Belgium; ^2^Institute of Computer Engineering, University of Lübeck, Lübeck, Germany

**Keywords:** decision-making process, multi-agent system (MAS), swarm robotics, collective system design (CSD), information processing

## Abstract

Autonomous decision-making is a fundamental requirement for the intelligent behavior of individual agents and systems. For artificial systems, one of the key design prerequisites is providing the system with the ability to make proper decisions. Current literature on collective artificial systems designs decision-making mechanisms inspired mostly by the successful natural systems. Nevertheless, most of the approaches focus on voting mechanisms and miss other fundamental aspects. In this paper, we aim to draw attention to the missed pieces for the design of efficient collective decision-making, mainly information processes in its two types of stimuli and options set.

Autonomous decision-making is a fundamental requirement for the intelligent behavior of, both, individual agents (Russell and Norvig, 1995) as well as collective systems (Mallon et al., 2001; Nicolis and Dussutour, 2008). In nature, there is a wide range of scenarios, from simple individual decisions about the direction of movement to more complex group decisions, that demonstrate the importance of achieving an appropriate decision-making, for example, selecting a new nest site in social insects (Mallon et al., 2001). Instead, we focus on designing collective decision-making processes in the context of artificial systems. We define this process as the emergence of a particular decision at the system level based on the opinions formulated and exchanged among its individuals (i.e., agents), who interact continuously and influence the opinion of each other. We restrict our study to “liquid brains” (dynamic network of agents moving in space) instead of “solid brains” (Gold and Shadlen, 2007; Pinero and Sole, 2018).

As a modeling assumption to help analyzing the system dynamics, we require the collective decision-making process to be discrete in both, the decision options and time, such that there are identifiable phases, for example, before and after an option was found/taken. An example of a system that we do not cover here is the Boids model of collective motion by Reynolds (1987), where the option space is continuous and it may never be clear when the swarm reconsidered to take a different direction. A borderline case is the behavior generated by the BEECLUST algorithm (Schmickl and Hamann, 2011) where the initially continuous option space (physical space) collapses by the agents into discrete options of competing clusters option space.

Collective decisions are required to be coherent, that is, by relying on common information and reaching consensus, it allows the system to act as one entity when confronted with different inputs and stimuli (Zabzina et al., 2014). Generating processes that lead to coherent decisions are subject to a tension in the decentralized system between (i) the individual freedom of choosing actions and (ii) the common goal of the system. Despite this tension, many natural systems illustrate how a collective system can make self-organized and coherent decisions. Typical examples include social insects (Sasaki and Pratt, 2017), neurons of a brain (Reid et al., 2015), and cells of the immune system (Shin and Mahrou, 2014). We take inspiration from the efficient decision-making demonstrated by natural collective systems to extract a complete description of requirements for decision making in artificial collectives. Natural systems address all the phases of a decision-making process, in addition, they learned to be adaptive and survive in most real-world scenarios. By analyzing these systems carefully, we can find a structured approach of collective-decision making for technical systems, such as swarm robotics (Hamann, 2018b) and self-organizing multi-agent systems (Wen et al., 2016), artificial immune system (Timmis et al., 2004), and future hybrid societies (Hamann et al., 2016). We identify different phases in these processes.

- Sense the need to start a decision-making process: In this phase a trigger that is defined by a particular set of stimuli at the system (macroscopic) level needs to be perceived at the individual level.
- Explore available options for the decision: This phase starts at the individual level and emerges at the system level, that is, the whole system needs to become aware of possible alternatives—the options. The potential decision options are identified in an exploration process, which is performed by the system individuals. The resulting set of options is strongly dependent on different parameters of the exploration process including the exploration space (range), the coverage of the space, the system size, and speed (e.g., deadlines) associated with the exploration process. In addition, the options depend on the features and capabilities of the individual agents, mainly their sensory capabilities.
- Achieve a coherent decision: Once the set of available options has been identified, in a final phase the collective system has to find a consensus on one option, and hence act as a single unit (Yu and Wang, 2010).

While we identify these phases for designing artificial systems, they also correspond quite well to a common subdivision of human decision-making by Orville et al. (1962). The authors define five phases, with the first starting from the identification of the problem followed by phases of obtaining information, producing possible solutions, and evaluating them. The production and evaluation of solutions translate here to the identification of options and to the assessment of their quality. Whereas, the first two phases of identifying the problem and gathering information translate to sensing the need for starting a decision-making process. The last phase defined by Orville et al. (1962) is selecting a strategy which corresponds to reaching consensus in a collective system.

Research on designing collective decision-making mainly investigates the relationship and interplay between two components: (i) the individual (microscopic) level, and (ii) the system (macroscopic) level. A third component, however, is often treated implicitly: information in the form of perceived stimuli and explored decision options. Here, we argue for the thesis that researchers should dedicate more effort to investigate and integrate the initial triggers of collective decision making, aiming to unravel the underlying principles and mechanisms to sense the need to start exploring available options. By highlighting the required aspects to perceive stimuli and learn about alternative options that are understudied across the different levels of the decision-making process, our main goal is to find a more structured and realistic approach for efficiently designing collective decisions in artificial systems. With this paper, we hope to create awareness of the missing pieces that are fundamental for a detailed investigation in future research of artificial systems. The arguments of our analysis are grounded in both biology as inspiration and robotics as a use case.

A prominent example of a robotic collective system is swarm robotics, a scalable approach of coordinating large groups of robots (Hamann, 2018b). Robot swarms are often used as artificial systems to analyze and validate collective decision-making approaches in dynamic complex systems (Trianni and Campo, 2015; Valentini et al., 2016). They take inspiration from natural collective systems, that address all relevant decision-making phases and survive successfully within their real-world scenarios. In contrast, collective decision-making in robot swarms can be demonstrated in an artificial context that takes mainly care of the phase, in which proper voting mechanisms and interaction models are defined for the decision process, without providing solutions to any of the other phases mentioned above. Given the early stage of swarm robotics research and the temptation to focus exclusively on the actual decision-making process, we find that especially the two initial phases of decision making—i.e., sensing the need to start and exploring available options—have often been ignored (totally or partially). We analyze the different aspects, to which we would like to draw more attention, in light of the three levels associated with any collective decision-making process as illustrated in Figure 1. In this figure, we show by color-coded features how the sensitivity of individuals determines the influx of decision information and in turn influences the characteristics of the emergent behavior at the system level.

**Figure 1:**
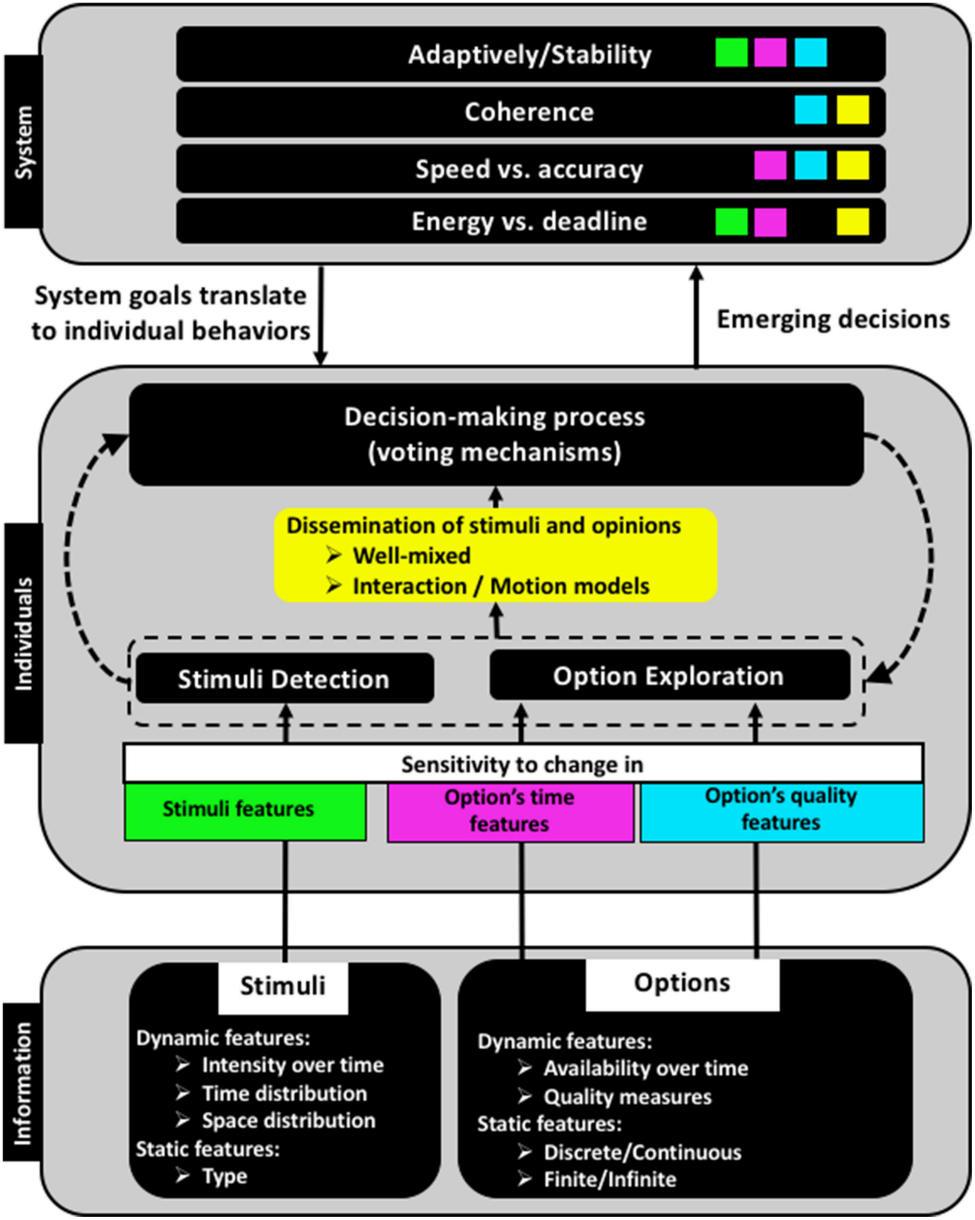
Three conceptual levels of collective decision-making and their interactions. The color scheme shows how properties of the available information and of the individual agents influence system-level features.

## 1. The Neglected Pieces of the Decision Process: Stimuli and Options

For collective decision-making, we highlight two important types of information that need to be acquired by the system and we refer to those as the neglected pieces of the decision process: (i) stimuli, and (ii) the set of options (alternatives) available for a particular decision (Couzin et al., 2005). A stimulus is the signal that triggers the system to start a decision-making process. The system's change in majority opinions is then its response to that stimulus. Different characteristics of stimuli and options, such as their rate and their spatial distributions influence the output of the decision process fundamentally. Nevertheless, characterizing this information and analyzing its consequences is mostly ignored in the literature of collective decision-making in artificial systems. In the following, we discuss the two types of information and some of their influential characteristics.

### 1.1. Decision Stimuli

Before a decision is after a decision. In general, the current state of the system is achieved as a result of a previous decision-making process, that led the system to select this particular state based on a set of given conditions. Hence, any change in these conditions (stimulus) can trigger the system to switch to another state.

The need of starting a decision-making process thus depends on a certain stimulus that is perceived either by a group of individuals or by the swarm collectively. A problem that is seldom studied is on recognizing the system's need to undertake decision-making processes. Indeed, studies on artificial systems (e.g., in robot swarms) often make the implicit assumption that there is system-wide awareness of the need to start a decision-making process. Usually, studied decision-making scenarios start at that moment when the system is assumed to have already gained global awareness about the need to start the collective decision-making process. Hence, mainly the agreement mechanisms are investigated in terms of their application at the individual level, as well as their emergent effect at the system level (Hamann et al., 2014; Valentini et al., 2014; Strandburg-Peshkin et al., 2015).

Inspired by natural systems, stimuli can be of two types: internal and external. Internal stimuli are associated with a change in system (or individuals) needs (e.g., a growth in the populations size, see Britton et al., 2002; Seeley, 2010, or in robots' on-board energy, see Khaluf and Dorigo, 2016). External stimuli are associated with a change in features of the system's current state, such as environmental changes, for example, a change in the intensity of the heat at the aggregation spots of honeybees (Szopek et al., 2013).

Individuals in natural systems capture both internal and external stimuli driven by their ultimate goal of surviving. Differently, artificial systems are designed to satisfy a set of goals, which may change according to their application. Such dynamic conditions make the identification of a specific set of stimuli—that lists the changes upon which the system should trigger a decision-making process—a critical challenge (Ashikaga and Asgari-Targhi, 2018). This challenge is not only related to the type of stimuli to which the system needs to react, but also to optimize the size of such a set when defined. Designing the system to react to a large number of stimuli has the risk of the system becoming vulnerable to fluctuations that result from developing several responses to a large number of changes. In addition, large stimuli sets can introduce conflicts in the system's reactions, that require to be resolved before the system can converge to a proper response. Hence, the stimuli set needs to be minimized carefully since missing particular stimuli reduces the system's adaptivity.

Once the stimuli set is defined, the next challenge is to design the individual mechanisms that guarantee a proper detection of the defined stimuli. This belongs to the micro-macro-link challenges in collective systems: deriving individual rules from a global goal (i.e., the particular stimulus) and vice versa. This challenge brings along a list of additional research questions, such as how to define the sensory capabilities of agents (Rodrigues et al., 2015; Salva et al., 2015) and how to decide between homogeneous or heterogeneous individuals in terms of detection mechanisms and the associated costs. In case of heterogeneous individuals, the size of the different sub-populations and their spatial distribution needs to be investigated.

### 1.2. Decision Options

Without options, there is no decision to take. Once the system has recognized its need to take a decision, the next step is to explore the set of possible options in order to select either the best option or to find a good-enough option within a limited time.

The set of options can be discrete, for example, in an aggregation scenario at predefined positions in space (Campo et al., 2011) or continuous, for example, when selecting a common direction of travel (Salge and Polani, 2011; Sartoretti et al., 2014). Additionally, the number of options can be finite, for example, as in a symmetry-breaking binary decision problem (Zabzina et al., 2014) or infinite, for example, choosing the velocity vector in flocking (Santos et al., 2016). We also distinguish between a static option set, where the options are known a priori, and a dynamic option set, where a subset of options is known in the beginning and options evolve over time (evolutionary option set). For a static set, the main challenge is to efficiently explore the quality of each option, whereas, for dynamic option sets, the challenge is to ensure the ability of the system to continuously explore and reveal new alternatives.

Generally, options are not of the same quality—problems which deal with same-quality options are referred to as symmetry-breaking problems. Measuring the quality of the available options is a design requirement and challenge. It is a requirement since the quality of a particular decision depends highly on the quality of the selected option. It is a challenge since most works assume the individuals of collective systems are able to perform noise-free assessments of option qualities, that is, an objective assessment of quality exists and is agreed upon among the different individuals in the swarm (Shackleton et al., 2016; Valentini et al., 2017; Hamann, 2018a). Only recently, research started to tackle this rather non-realistic assumption, as discussed by O'Shea-Wheller et al. (2017) and Richardson et al. (2018), where the influence of the differences in the assessments at the individual level was analyzed and reported in terms of the resultant decision-making process. For a realistic design of decision-making processes, we need to consider sensor noise and to include it in the models of individual agents of the collective system. This may increase the complexity of how to find consensus over the options qualities. Furthermore, we need to design our individual behaviors such that exploration for new options is optimized and option detection mechanisms are integrated.

## 2. From the Neglected Pieces to Dissemination and Decision-Making

In order to decide, the system needs to become aware of both its stimuli and options. For stimuli, the information flow is passively triggered through the perception of a subpopulation. Individuals do not explicitly explore to gain information about stimuli, instead, they merely receive information about changes in their system or environment, once they occur. Options, in turn, represent potential solutions that can be adopted in a response to changes in the system's conditions triggered by specific stimuli. Hence, the information about the available options is gained actively through an exploration process of the individual agents. Consequently, designing the individual behavior to demonstrate efficient detection and exploration behaviors is a crucial prerequisite for a successful aggregation of the information required for the decision-making process. Once these pieces of information have become available, two steps follow: First a fair dissemination (sharing) of the information; secondly, a coherent voting mechanism to select one of the discovered options.

### 2.1. Exploration

In artificial systems, the literature on collective decision-making is mainly focused on voting mechanisms used to select a particular option and consecutive effects. Whereas, the prior exploration process that led to the identification of these options is often ignored. It is mostly assumed that individuals have a priori knowledge of options or at least know how to navigate in space to discover available options, as observed in natural systems such as bees (Menzel et al., 2005). However, individuals in artificial systems need to be equipped with well-designed mechanisms and tools to navigate through problem space and to detect options, and furthermore infer their qualities. The efficiency of searching strategies is application-dependent and requires well-adapted exploration trajectories for a given distribution of options in space (Bartumeus et al., 2005). This is challenging since (i) the distribution of options in the problem space is unknown, and (ii) the system is associated with a set of limitations, such as system size, travel speeds, sensor coverage, time per measurements, and available total time (Kao and Couzin, 2014). According to the nature of robotics systems and their applications, the most critical limitations are due to limited time, that is, deadlines for decisions (Khaluf et al., 2014, 2016) and limited energy, that is, available energy at the individual level and the amount that can be allocated to the exploration process (Ratnieks and Shackleton, 2015; Wolf et al., 2016). Therefore, more efforts need to be dedicated to the design of efficient exploration strategies (in term of time, energy, and coverage) to allow the emergence of more adaptive and accurate decision processes.

### 2.2. Information Dissemination and Well-Mixed Systems

Once any piece of decision-related information (i.e., stimulus or option) becomes available for a subset of the individuals in a collective system, the next challenge is to make the whole system aware of this information in order to prepare for an appropriate response. Sharing information in collective systems is realized by both direct and indirect interactions among the agents (Pitonakova et al., 2016; Meyer, 2017). Direct interaction refers to individual-to-individual interactions. Whereas, indirect interaction refers to communicating information through the environment, using techniques such as stigmergy, e.g., pheromone trails in foraging ants (Dorigo et al., 2000). In the context of decision-making processes, direct communication is the most investigated model for interactions, in which the knowledge of an individual agent is communicated by local interactions at a shared central place (e.g., nest as observed in honeybees (Seeley et al., 2012; Reina et al., 2018). Local interaction models allow the collective system to be scalable since its dynamics emerge from the information shared in the neighborhood of each individual and no central component is needed. However, other interaction models were also observed in collective biological and physical systems, such as scale-free correlations and networks (Cavagna et al., 2010; Hemelrijk and Hildenbrandt, 2015) or small-world networks (Hlinka et al., 2017). These models were claimed to lead to significantly better results concerning decision coherence and response time.

In general, the interaction model influences mainly two features of collective decisions; (i) the degree of decision coherence, that is, the percentage of individuals that are committed to the same opinion (Khaluf et al., 2017a), and (ii) the speed of decision-making via the propagation speed of information in the system (Sumpter et al., 2008). Another individual parameter that plays a main role in how to improve information sharing is the spatial distribution of information, which is defined indirectly by the motion pattern and density of agents (Stradner et al., 2013; Khaluf et al., 2017b). These two parameters influence how well-mixed the system is, that is, whether each individual has an equal chance to interact with any other individual. A well-mixed system state and how to get there quickly facilitates reachability of decision-related information in the collective system (Torney et al., 2015). Therefore, designing individual motion patterns with respect to resulting information flows and densities is a key challenge.

### 2.3. Voting Mechanisms

The emergent decision at the system level is essentially affected by the voting mechanism that is applied by the individuals. Voting mechanisms describe the logic of commitment to a particular option. These mechanisms are obviously highly relevant and have been intensively studied across the literature of decision-making in artificial collective systems. Hence, they are of lower interest here. However, it is still important to notice that some system characteristics such as the density of the collective system or the underlying interaction model influence the usefulness of a particular voting mechanism. For example, using the majority rule as decision-making mechanism (Scheidler et al., 2016) is difficult in sparse systems (Khaluf et al., 2017b). Consequently, during the design of a collective decision-making process, it is not only the selection of a voting mechanism but also the verification of effective conditions at the individual level, that then enables a successful application of that voting mechanism. Another understudied aspect is to provide the collective system with capabilities to switch between different voting mechanisms based on current conditions (e.g., neighborhood densities, information about available options, etc.). Currently, when designing collective artificial systems, often a voting algorithm is assigned to the system before analyzing the emergent decision-making dynamics (Kanakia et al., 2016).

## 3. The Emergent System Measures

The system level is the upper level at which the opinions adopted by the individuals—based on their collected information and interactions—emerge to form a united and coherent entity. As mentioned above, the main goal of this paper is to draw attention to missing pieces in designing collective decisions. Accordingly, we highlight a key set of system features and show how handling of stimuli and options is essential to achieve these.

### 3.1. Decision Coherence

The coherence of a collective decision describes the degree of agreement in the system, that is, the percentage of individuals who commit to a majority opinion. Coherence is improved by sharing information efficiently, that depends on the interaction model (as discussed in section 2), the motion model and the density of the system (see Figure 1). Particular interaction models, such as scale-free models, were found to result in high coherence. Similarly, some motion patterns, such as flocking, show a coherent response to stimuli, for example, in predator attacks (Romanczuk et al., 2009). Another influence on coherence is the individual's sensitivity to assess qualities of the available options (see section 1.2). This sensitivity influences whether a consensus (or alternatively a good enough coherence) is achieved about the option quality, which is fundamental for the subsequent decision-making process.

### 3.2. Speed vs. Accuracy

A collective system can either decide fast or accurately. Another important quality of a collective decision is associated with the well-known trade-off between speed and accuracy. This challenge exists already for static option sets (see section 1.2) but gets more relevant for options evolving over time (Franks et al., 2003). Deciding fast about the known set of options at a given time may limit the accuracy of the decision-making process even more so because better options may appear later. This also relates to the more general secretary problem (Freeman, 1983). In light of the speed-vs.-accuracy tradeoff, we see how important it is to choose an appropriate mechanism to handle and share decision-related information. For example, a high sensitivity to changes in temporal or qualitative features of options may lead to more accurate decisions (by accounting for all options' updates), but slows down the convergence to a stable decision. Similarly, relying on efficient interactions and well-mixed system states can accelerate the convergence to a decision, but may miss some available options and hence limit accuracy (see Figure 1). A useful technique is to postpone the decision-making process if the quality of the currently available options is too low and hence taking the risk of waiting for better options (Freeman, 1983; Reina et al., 2017). This family of decision-making processes is referred to as value-based decision making (Pirrone et al., 2014). A large list of works cover both types of option sets. However, often it is assumed to be a static set that is known a priori. This assumption doesn't hold for unknown environments that our artificial collective systems will face in applications.

### 3.3. Adaptivity and Stability

Once decided, when to reconsider? A key feature in designing artificial systems is adaptivity. Adaptivity represents the ability of the system to modify its state as a response to particular changes. The quality of the system's state may have changed making the current state less desirable than before or a new state may have emerged that is more desirable than the current state. In both cases, an adaptive system would switch to the most appropriate known state. To implement adaptivity, revising the latest decision (state) is necessary. In the literature, the most common approach is by using noise at the individual level to derive decision revisions (Balázsi et al., 2011; Biancalani et al., 2014; Hamann et al., 2014; Khaluf et al., 2017b). This approach allows individuals, who are committed to a particular option to switch their opinion spontaneously, and may convince their neighborhood to switch opinion, too. A cascading effect may impact the global level and switch the system's current state. For example, the authors in Ioannou et al. (2011) call for new mathematical approaches to help attaining insights into how information about potential options needs to be acquired in a collective system in order to build a proper response to specific stimuli.

In the context of dynamic collective systems, adaptivity needs to be balanced with stability. Stability can be defined in terms of the rate at which the system changes its current state. High stability corresponds to a low rate in switching states of the observed systems, while a poor stability corresponds to a high switching rate. Changes that require the system's response can occur to both stimuli and option sets. However, not every change can be addressed nor should the system react to each change. High rates of changes in the stimuli may drive the system to instability and may keep the system in unproductive transient states. Despite its intuitive importance, stimuli rates are seldom discussed in research works on the design of artificial collective systems. One notable exception is made by Herbert-Read et al. (2015) who designed a flocking behavior where consensus on a specific velocity should be achieved before a deadline in order to avoid a predator attack (i.e., a hard deadline). Therefore, it is a fundamental requirement when designing the individual behavior to define an appropriate sensitivity threshold (see Figure 1) to respond to any kind of stimuli information. Hence, a reasonable balance between adaptivity and stability can emerge without driving the system into chaos (i.e., disorder) (Strogatz et al., 1994). One approach is to provide enough positive feedback, such that the system can still switch opinions and to balance the rate of spontaneous switching at the individual level (high individual noise), which functions as negative feedback and hence keeps the system in an undecided state (Khaluf et al., 2017b).

## 4. Conclusion

We have discussed a key process in artificial collective systems: decision-making. Decision-making is fundamental to obtain autonomy and is an essential building block of artificial intelligence. Similarly, collective decision-making implements autonomy on the global level for collective systems and introduces an interesting two-level autonomy with a micro- and a macro-level. We have highlighted key but neglected pieces of collective decision-making processes. We argued that without addressing

- how a collective system locally detects that a collective decision is required (stimuli),
- and how a collective system explores and disseminates potential alternatives (options)

we will not be able to engineer autonomous collective systems that survive in the real world.

The requirement for a decision is perceived via certain stimuli, of which the detection and decoding are major challenges. Hence, the individuals of an artificial collective system need to have both efficient algorithms and proper hardware to process specific information of two forms: internal stimuli (e.g., change in system needs) and external stimuli (e.g., dynamic environments).

The second main challenge is the exploration and detection of potential options. Often in the literature of collective decision-making in artificial systems, the system's individuals are assumed to explore efficiently and being able to identify options when encountered. Nevertheless, gathering information about potential options is a fundamental problem that is directly related to changes occurring in system states. Depending on the type of changes, different sets of options need to be identified. For example, having low energy levels must lead the individuals to explore for energy sources instead of shelter alternatives. Additionally to address properly option challenge, the individual behavior needs to guarantee an efficient and continuous exploration of the problem space in order to discover unknown and revisit known options (if still valid).

For the successful design of collective decision-making, engineers need to fully consider the individual mechanisms that are used to perceive decision signals via stimuli, to decode and process these, and to adopt proper exploration and identifying strategies to address options. Then the efficient dissemination of that information in addition to proper voting mechanisms is a key to a converging and adaptive decision-making process.

Our overall objective is to help guiding a complete design process of collective decision-making for artificial systems, such as in swarm robotics. Future research has to focus on these understudied aspects to develop an efficient methodology for all different phases of collective decision-making. This way we will be able to prepare sufficient degrees of autonomy for our future artificial collective systems in real-world applications.

## Author Contributions

YK conceived and wrote the first version of the paper. YK and HH discussed and defined the main research questions and the critical missing elements in the current design of a collective decision-making process. YK and HH have analyzed the role the different individual mechanisms play in shaping the emergent system features under a collective decision-making process. YK and HH have set up the overall structure of the paper. YK, HH, and PS have converged over several review rounds at the final structure and outline of the paper. PS reviewed the final versions for clarity, consistency, and style. YK coordinated the work of all coauthors.

### Conflict of Interest Statement

The authors declare that the research was conducted in the absence of any commercial or financial relationships that could be construed as a potential conflict of interest.
